# Can We Improve the Monitoring of People With Multiple Sclerosis Using Simple Tools, Data Sharing, and Patient Engagement?

**DOI:** 10.3389/fneur.2020.00464

**Published:** 2020-06-12

**Authors:** Kimberley Allen-Philbey, Rod Middleton, Katie Tuite-Dalton, Elaine Baker, Andrea Stennett, Christo Albor, Klaus Schmierer

**Affiliations:** ^1^Clinical Board Medicine (Neuroscience), The Royal London Hospital, Barts Health NHS Trust, London, United Kingdom; ^2^UK MS Register, Swansea University Medical School, Swansea, United Kingdom; ^3^The Blizard Institute (Neuroscience, Surgery & Trauma), Queen Mary University of London, London, United Kingdom

**Keywords:** multiple sclerosis, monitoring, 3TEST, patient engagement, technology

## Abstract

Technological innovation is transforming traditional clinical practice, enabling people with multiple sclerosis (pwMS) to contribute health care outcome data remotely between clinic visits. In both relapsing and progressive forms of multiple sclerosis (MS), patients may experience variable disability accrual and symptoms throughout their disease course. The potential impact on the quality of life (QoL) in pwMS and their families and carers is profound. The introduction of treatment targets, such as NEDA (no evidence of disease activity) and NEPAD (no evidence of progression or active disease), that guide clinical decision-making, highlight the importance of utilizing sensitive instruments to measure and track disease activity and progression. However, the gold standard neurological disability tool—expanded disability severity scale (EDSS)—has universally recognized limitations. With strides made in our understanding of MS pathophysiology and DMT responsiveness, maintaining the status quo of measuring disability progression is no longer the recommended option. Outside the clinical trial setting, a comprehensive monitoring system has not been robustly established for pwMS. A 21st-century approach is required to integrate clinical, paraclinical, and patient-reported outcome (PRO) data from electronic health records, local databases, and patient registries. Patient and public involvement (PPI) is critical in the design and implementation of this workflow. To take full advantage of the potential of digital technology in the monitoring and care and QoL of pwMS will require iterative feedback between pwMS, health care professionals (HCPs), scientists, and digital experts.

## Introduction

Multiple sclerosis (MS) is a chronic inflammatory, demyelinating, and degenerative disease of the central nervous system (CNS). MS affects more than 130,000 people in the UK and over 2.5 million worldwide (1–3). While prediction of the disease trajectory in individual people with MS (pwMS) remains challenging, accrual of chronic disability is the norm (4, 5), particularly if pwMS are left without disease-modifying treatment (DMT) (6). Dependable outcome measures are highly desirable to assess the clinical course of MS and inform patient management. Given the heterogeneity of clinical presentation, systems involved, and speed of progression, assessing outcomes in pwMS requires systematic, multidimensional tools. Comprehensive follow-up of pwMS has been demonstrated in a number of clinical trials (7–10). However, systematic monitoring of pwMS in clinical practice is often incompatible with the limited time available for patient review (11), particularly when using the expanded disability status scale (EDSS) (12), which nevertheless remains key to determine DMT eligibility (13), and despite its well-rehearsed shortcomings (14).

PwMS with advanced disease, for example those having an EDSS ≥ 6.5, and elderly pwMS are at particular risk of being less carefully followed up (15). These patients are more likely not on a licensed DMT and are commonly considered “beyond” immunotherapy, despite mounting evidence that neurologic function can potentially be preserved, even at a later stage of the disease (16, 17).

Here, we provide a perspective on using a new approach of collecting data in pwMS that combines (i) clinical assessments with potential for self-monitoring and (ii) patient-reported outcomes (PROs) using a platform shared between a large data repository, the UK MS Register at Swansea University, and BartsMS in east London, UK. We describe how such point-of-care data collection may serve both research and the individual pwMS in clinic and highlight the role of patient and public involvement (PPI) in facilitating the “buy-in” of pwMS underpinned by some preliminary data on patient engagement with the UK MS Register portal and corresponding data sharing preferences.

## Quantifying Neurologic Disability

The introduction of the EDSS (12) as the key outcome measure of disability in MS DMT trials cemented its role as the neurologist's “gold standard” rating scale of disability in pwMS. However, while clinical trials usually allocate sufficient time to complete and fully document an EDSS (which takes ~20–30 min), the time constraints of clinical practice regularly lead to either an “estimated” EDSS, or systematic clinical assessments remain patchy, or are not undertaken at all (11). To overcome this shortcoming, various versions of a patient-reported EDSS (PREDSS) have been proposed. These are either paper based, administered via telephone, or, more recently, via an online application, the “webEDSS” (18). Correlation has been observed between EDSS and all versions of PREDSS; however, limitations of agreement were identified, particularly at low EDSS levels (11). However, even if these limitations could be minimized, the non-parametric character of the EDSS, its ambulatory bias, and lack of sensitivity at high values remain problematic. Moreover, decline in cognitive function is not well covered, in spite of its key importance in pwMS, especially given the implications for employment opportunities (19, 20).

As a result, the National MS Society's Clinical Outcomes Assessment Task Force started more than 25 years ago developing a new set of outcome measures. Ultimately, a set of three tests was agreed, making up what was coined the Multiple Sclerosis Functional Composite (MSFC). The MSFC consists of the Paced Auditory Serial Addition Test (PASAT), Timed 25-foot walking (T25ftWT), and the Nine Hole Peg Test (9HPT) and has been implemented in a number of clinical trials (21). However, only this year, 2020, will a DMT licensing trial for the first time use one element of the MSFC, the 9HPT, as its primary outcome measure (22).

“BartsMS” is a clinic–academic partnership based at The Royal London Hospital (Barts Health NHS Trust) and The Blizard Institute/Queen Mary University of London providing clinical care to over 3000 pwMS. Faced with the same discrepancy between high expectations and the reality of limited resources (6), BartsMS introduced a modified version of the MSFC in their clinical practice in 2016. While T25ftWT and 9HPT were retained, PASAT was replaced with the Symbol Digit Modality Test (SDMT; oral version) following the recommendation by Drake and coworkers (23), among others (24). The SDMT has equal psychometric validity to the PASAT and is associated with lesser confounding by training and more congenial for both patient and assessor (23). It takes less time to complete, requires less expertise and experience of the assessor, and, unlike the PASAT, does not require special equipment for auditory presentation of stimuli (24). In practice, we summarize the three elements (T25ftWT, 9HPT, and SDMT) simply as “3TEST.” Given a clinical and research focus of BartsMS on advanced MS, i.e., people with an EDSS of ≥ 6.5 (25), the ABILHAND questionnaire is also regularly administered to capture perceived manual ability (26). Obtaining such “real world” outcome measures in routine clinical practice and trials has also been recognized by the European Medicines Agency (EMA) as an important component of disease management (27).

## The Evolving Role of Remote Self-Monitoring

The relative simplicity of the MSFC or variations thereof, such as the 3TEST, combined with advances in technology and ever-increasing online resources and capabilities have led to the expansion and uptake of self-monitoring applications (28). Self-monitoring enables tracking the disease course in pwMS unable to travel to clinic, e.g., due to their disability or them living in remote locations. Given the often-extended intervals between follow-up in clinic (commonly 6–12 months), systematic self-monitoring may improve detection of changes not captured during visits, including relapses and disability accrual, thereby enabling earlier detection of disease progression and trajectories of long-term outcomes. Moreover, self-monitoring has inherent potential to empower pwMS to manage their condition pro-actively, with likely benefits for their care and self-management (29). Alongside other measures, such as written decision aids (30), self-monitoring may help remove hierarchical barriers and level the platform for shared decision-making between health care professionals (HCPs) and pwMS. It would be expected that such change will improve treatment satisfaction and adherence (31). Against this backdrop, numerous self-assessment tools have been developed (32, 33). As part of this effort, our group developed portable versions of the 9HPTs and the T25ftWTs (34, 35), while the UKMSR produced an online version of the SDMT (MSiDMT) (36).

In addition, wearable technologies, including motion detecting devices (MTDs) and smartphone applications may facilitate minimally intrusive assessment of outcomes such as step count, walking speed, and gait (37) and support neurorehabilitation (38).

## A Model of Integrated Monitoring and Patient Engagement

Results from tests that (i) are relatively straightforward to implement in clinic and (ii) can be translated into self-monitoring tools can be combined with PRO questionnaires and fed into the patient record, which, in health care settings covering large numbers of pwMS, is usually an electronic health record (EHR). EHRs facilitate the timely recording of patient data and the simultaneous navigation by multiple HCPs from different specialities (39). Coding terminology, such as Systematized Nomenclature for Medicine (SNOMED), provides a powerful resource to collate individual patient data as well as to identify, stratify, and audit patient cohorts and outcomes.

We use the generic Barts Health NHS Trust-wide EHR Cerner Millennium Clinical Record System (CRS). This system enables extraction of coded information to populate our database of pwMS (the “BartsMS Database”) in Excel (40), thereby providing both an individual record and a point-of-care data collection, including 3TEST data, fed by the various HCPs at the Trust involved in the care of the pwMS. Our dataset is further enriched by the UK MS Register (UKMSR), an MS Society (UK)-sponsored resource that collects PRO data on pwMS throughout the UK (41). The UKMSR was conceived on the understanding that PRO data are important to capture the experience of pwMS and their families, friends, and carers (42–44). PROs are also commonly used as secondary endpoints in clinical trials to determine and compare the effect of DMTs. The core validated instruments collected by the UKMSR are EuroQol 5D (EQ-5D), Multiple Sclerosis Impact Scale 29v2 (MSIS-29), Hospital Anxiety and Depression (HADS) Scale, Fatigue Severity Scale (FSS), the Multiple Sclerosis Walking Scale (MSWS-12), and Patient Determined Disease Steps (PDDS) (45–51). The webEDSS is also available as an *ad hoc* questionnaire (52).

**Figure 1:**
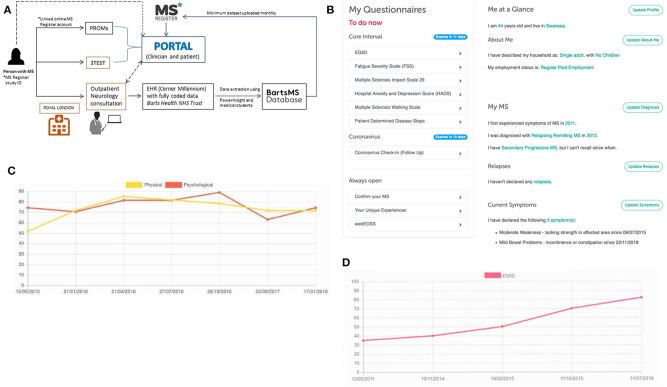
The hub/spoke system between BartsMS and the UK MS Register. **(A)** The interaction between the BartsMS service & Database, and the UK MS Register. **(B)** My MS Hub page on the patient portal. **(C)** Graph of Multiple Sclerosis Impact Scale (MSIS29 V2) results viewed in the patient portal. Accompanying description for people with MS: “Used to measure the impact that your MS is having on you physically and psychologically at any given time. This questionnaire was designed specifically for MS. It means that researchers can measure how much your MS affects your quality of life. It is increasingly being used in MS research and clinical settings. What the graph means: the MSIS is composed of three scores, a total, a psychological sub-score and a physical sub-score. In this graph we show the psychological and physical sub-scores. In both Scales, higher values indicate that your MS is causing you more trouble and lower ones indicate less impact.” **(D)** Graph of EQ-5D 3L results viewed in the patient portal. Accompanying description for people with MS: “This is one of the most commonly used health status measurements. It is not MS specific and so it can be used to make comparisons with various other health conditions/chronic diseases. What the graph means: the EQ5D has five questions specifically related to aspects of your general health and one scale marked out of 100 in which you indicate your overall quality of life. High scores indicate a better overall quality of life”.

Since 2017, BartsMS and the UKMSR have been developing a hub/spoke monitoring system (Figure 1). The intention of the algorithm is to (i) facilitate research through high-quality data collection, (ii) support the clinical service provision with PRO data, and (iii) enable the latter via a patient portal. PwMS who consent to join the UKMSR will have their minimum dataset (demographics, MS history, risk factors, disease course, EDSS scores, relapses, DMT, and symptomatic information) collected and securely uploaded via a REDCap electronic clinical record form (53). In addition, pwMS are prompted via email, at regular (currently 6-monthly) intervals, to fill in PRO questionnaires. This information can then be linked to their unique study ID provided at the hospital site, and thereby merged with their clinical record.

## Patient Engagement

We learned that patient and public involvement (PPI) is pivotal to maintain and expand data collection through the UKMSR. Valuable insights and feedback were provided through a PPI meeting held at The Royal London Hospital (Barts Health NHS Trust) on 16 February 2018. Key outcomes of this engagement day were (i) a re-designed, visually more attractive website enabling easier navigation and providing better sectioning, including a “My MS” hub page. This hub contains easily identifiable and accessible open questionnaires, including estimates of the time required for completion. This feature also provides pwMS with a snapshot of the information they have contributed and highlights any data that they should still provide; (ii) radio boxes for questionnaires, rather than drop-down menus since less mouse movement is required, making it easier to navigate for pwMS with upper limb function impairment; (iii) reduced frequency of questionnaire responses requested (bi-annually instead of quarterly); (iv) more tangible benefits for UKMSR subscribers, who were keen to receive comprehensive feedback about their collected questionnaire data—we therefore decided that the facility of viewing personal response data should be provided as an option; (v) since September 2018, participants who join the UKMSR and opt in to feedback are being offered a downloadable version of their results. By December 2019, 67% of new subscribers (total n = 2712) had had opted into this facility. This is designed so that it can be taken along to clinic appointments. Information is displayed in easily accessible graphs, allowing pwMS to track their condition over time. Explanations in lay terms are included about what the instruments and graphs mean and their relevance to pwMS (Figures 1C,D).

Further insights from our PPI exercise included an understanding that pwMS wanted the UKMSR portal to enable them (i) to have better control over their health care including treatment options, (ii) access to clinical trials, and (iii) improved self-management. PwMS were also passionate about furthering research both for short-term benefit and for future generations, including their own children.

To estimate the effect of our response to the PPI input received on the rate of questionnaires, we extracted the number of completed questionnaires at three time points; Winter 2018 (before implementation of the above changes to the portal), Spring 2019, and Winter 2019. Data were extracted from the UKMSR production databases running Microsoft SQL Server 2014.

**Figure 2:**
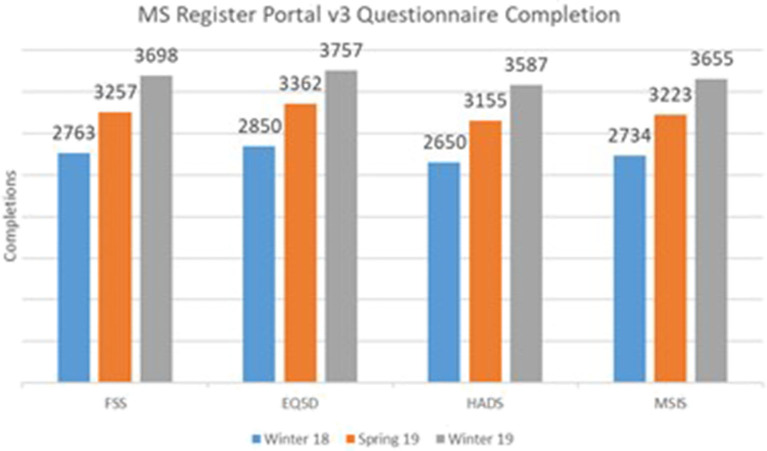
Core Questionnaire Response rates following PPI and redesign of the UK MS Register portal.

Figure 2 illustrates a significant increase in the number of completed questionnaires between the launch of the new website in Winter 2018 and the latest cutoff in Winter 2019. This increase suggests a significant impact of PPI on the new UKMSR portal design and functionality.

## Discussion

Optimizing the landscape of individualized, effective, and compassionate care with and for pwMS remains a work in progress. Whereas clinical trials provide data on a cohort level, the evidence produced can only provide a backdrop for decisions that need to be tailored to the individual pwMS. Clinical monitoring is essential to detect treatment success and failure, in order to make individual decisions. While various digital tools for disease monitoring in pwMS have been developed, their value in clinical practice is not yet established, and their adoption limited (54). We found validated measures that are easily applicable and straightforward to interpret a useful way to quantify change in an era where pwMS expect their care to catch up with the efficacy of the latest DMTs. The administration of 3TEST does not require any special qualification—virtually any HCP can be trained to apply it in a short timeframe. Since all three parts of the 3TEST can be done remotely, the limit for self-monitoring is now mainly a question of frequency and logistics (how often to test, how to feedback results to the health care team, and how to embed the data in the daily routine of neurologists and MS specialists between appointments). The simplicity and compatibility for remote testing of 3TEST also highlight the potential for relatively straightforward multi-center adoption and inclusion in large datasets, such as the UKMSR or MSBase (55), and there is obvious potential for remote testing in exceptional situations, such as a pandemic (56). Furthermore, 3TEST is likely going to be of use when screening for trials where measures other than the EDSS are being used for inclusion as well as outcome (22). New systems intended to both serve individual monitoring of pwMS and contribute to large datasets, such as Floodlight (33, 57), will need to be validated using well-established tests such as those combined in 3TEST (32).

Our experience trying to combine clinical and PRO data collection via the UKMSR in order to facilitate databasing for research, service audit, and individual patient care highlights the important role of PPI throughout the design and implementation process. To truly deliver patient-centered care and at the same time enable high-quality data collection, any system for pwMS needs to be developed jointly with pwMS. In our example, PPI led to a significantly increased number of completed PRO questionnaires. We are currently optimizing and streamlining mutual data exchange between BartsMS and the UKMSR to provide an integrate model of point-of- care data collection. This system may provide a model of data collection and sharing that can be adopted by other centers across the UK and beyond.

## Data Availability Statement

All datasets generated for this study are included in the article/supplementary material.

## Author Contributions

KS initiated the BartsMS Database and conceptualized the setup between the UK MS Register and BartsMS clinical interface, which the team helped establish. KA-P, RM, and KS drafted the manuscript. CA, AS, and KT-D contributed toward the subsequent revisions. RM and EB performed the data extraction and analysis. All authors read and approved the final manuscript.

## Conflict of Interest

The authors declare that the research was conducted in the absence of any commercial or financial relationships that could be construed as a potential conflict of interest. The handling editor declared a past co-authorship with one of the authors KS.
